# Prior exposure to an attenuated *Listeria *vaccine does not reduce immunogenicity: pre-clinical assessment of the efficacy of a *Listeria *vaccine in the induction of immune responses against HIV

**DOI:** 10.1186/1476-8518-9-2

**Published:** 2011-01-18

**Authors:** James B Whitney, Saied Mirshahidi, So-Yon Lim, Lauren Goins, Chris C Ibegbu, Daniel C Anderson, Richard B Raybourne, Fred R Frankel, Judy Lieberman, Ruth M Ruprecht

**Affiliations:** 1 Division of Viral Pathogenesis, Beth Israel Deaconess Medical Center, Boston, MA 02115, USA; 2 Harvard Medical School, Boston, MA, 02115 USA; 3 Loma Linda University Cancer Center, Loma Linda, CA 92354, USA; 4 Department of Cancer Immunology and AIDS, Dana-Farber Cancer Institute, Boston, MA 02115 USA; 5 Division of Research Resources and Microbiology and Immunology, Yerkes National Primate Research Center, Emory University, Atlanta, GA 30329 USA; 6 Immunobiology Branch, Center for Food Safety and Applied Nutrition, Food and Drug Administration, Laurel, MD 20708 USA; 7 Department of Microbiology, University of Pennsylvania, Philadelphia, PA 19104 USA; 8 The Immune Disease Institute and Program in Cellular and Molecular Medicine Children's Hospital Boston, Department of Pediatrics MA 02115 USA

## Abstract

**Background:**

We have evaluated an attenuated *Listeria monocytogenes *(Lm) candidate vaccine vector in nonhuman primates using a delivery regimen relying solely on oral vaccination. We sought to determine the impact of prior Lm vector exposure on the development of new immune responses against HIV antigens.

**Findings:**

Two groups of rhesus macaques one Lm naive, the other having documented prior Lm vector exposures, were evaluated in response to oral inoculations of the same vector expressing recombinant HIV-1 Gag protein. The efficacy of the Lm vector was determined by ELISA to assess the generation of anti-Listerial antibodies; cellular responses were measured by HIV-Gag specific ELISpot assay. Our results show that prior Lm exposures did not diminish the generation of *de novo *cellular responses against HIV, as compared to *Listeria*-naïve monkeys. Moreover, empty vector exposures did not elicit potent antibody responses, consistent with the intracellular nature of Lm.

**Conclusions:**

The present study demonstrates in a pre-clinical vaccine model, that prior oral immunization with an empty Lm vector does not diminish immunogenicity to Lm-expressed HIV genes. This work underscores the need for the continued development of attenuated Lm as an orally deliverable vaccine.

## Findings

More than 80% of new HIV acquisitions are through mucosal routes, underscoring the importance of generating HIV-specific immunity by vaccination at these sites [1]. A vaccine vector capable of inducing potent mucosal immunity would represent a promising candidate for development [2].

*Listeria monocytogenes *(Lm) is a ubiquitous intracellular bacterium that has served as a model inducer of innate and adaptive immunity to infection. Natural infection with wild-type *Lm *typically initiates via the oral route [3,4], and the breadth of immunity elicited by Lm, combined with a natural predilection for the gut has prompted their development as live vaccine vectors [2,4-7]. Lm vectors have been shown to be effective in both cancer [6,8,9] and in infectious disease settings [7,9]. Despite the attractive features of Lm vectored antigen delivery, there are potential obstacles to this approach.

Anti-vector immunity represents an important hurdle in the development of many recombinant vaccine-vector systems. For example, anti-vector immunity has been shown to markedly suppress the immunogenicity of replication defective recombinant Adenovirus-5 based strategies [10]. This problem has been circumvented using vectors that display hexon antigen from low seroprevalence subtypes, or boosting with different subtype vectors [10,11].

In the case of Lm, studies in murine and feline models have assessed the impact of anti-Listerial immunity on the generation of *de-novo *responses against Lm-expressed gene inserts [12-14]. To date, clinical studies have indicated that cellular immunity to Lm was present in approximately 60% of the cohort population [15]. Given the high likelihood of anti-Listerial immunity within the populations of both developed and developing nations [16], this issue is needful of further exploration.

In the current study, we update our progress on a *Listeria*-based candidate vaccine against HIV. We extend our immunogenicity studies by adopting a modified vaccine dose and delivery regimen relying solely on oral vaccination.

### Modified vaccine delivery

**Figure 1:**
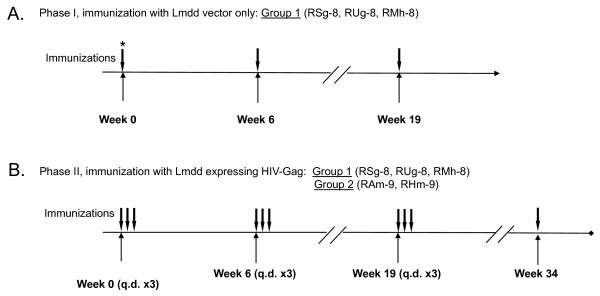
**Immunization schedule for administration of Lmdd or Lmdd-HIV-*gag***. A total of 5 individual monkeys were enrolled into 2 immunization groups: Group 1 (animals RMh-8, RSg-8, and RUg-8), received three oral inoculations of Lmdd empty vector alone during experimental phase I; the doses were 1 × 10^12 ^organisms at week 0 followed by 3 × 10^12 ^organisms at weeks 6 and 19 (vaccination shown as vertical arrows) (A). Group 2 (animals RAm-9 and RHm-9) were enrolled. In experimental phase II, both groups received Lmdd-HIV-gag orally in phosphate-buffered saline (PBS) at wks 0, 6, and 19 at 3 × 10^12 ^organisms given for 3 consecutive days (q.d. x 3) depicted in (B). *The dosage (in colony forming units/ml, CFU) administered at each time point is shown in parentheses for each group. All Lmdd-gag vaccinations were preceded by oral administration of saturated sodium bicarbonate. D-ala (640 mg/kg) was co-administered intravenously before and after each vaccine dose [17]. Lmdd inocula were also supplemented with D-ala (0.5 mg/ml in 20 ml) to ensure efficient bacterial replication.

Two groups of macaques, one previously exposed to the Lmdd vector (Group 1) and a Lm-naïve control (Group 2), were enrolled to test the immunogenicity of Lmdd-HIV-gag [17]. We sought to assess safety and immunogenicity after modifying the regimen to oral only delivery of Lmdd-HIV-gag over 3 consecutive days (q.d. x3) for priming and two consecutive boosts (Figure 1).

### Phase I: immunization with empty vector Lmdd

**Figure 2:**
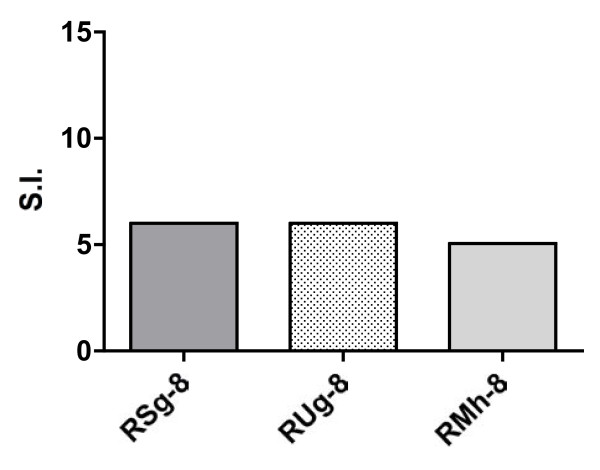
***Listeria*-specific proliferative responses in immunized macaques**. PBMC from individual monkeys were tested for *Listeria*-specific proliferative responses at the indicated time points after inoculation with the empty Lmdd vector. Cells were cultured in supplemented RPMI in the presence of HIV IIIB p55 Gag (2 μg/ml) for 4 d. Cells were pulsed with 1 μCi per well of ^3^H-thymidine (PerkinElmer, Boston, MA) for 18 h prior to harvesting. Thymidine incorporation was assessed using a β-scintillation counter (Beckman Coulter, Inc., Miami, FL). Results are expressed as stimulation index (SI). To test for Lm-specific proliferative responses, whole Lm bacteria (strain 12443) were used as described [17].

Group1 monkeys (RSg-8, RUg-8 and RMh-8), received Lmdd orally in conjunction with i.v. administration of D-ala (Figure 1A). Repeated oral immunization with empty Lmdd did not induce significant anti-Lm humoral immunity (data not shown). However, marginally significant proliferative responses (5-6 fold above background) were detected in response to stimulation with LLO peptides in all Group 1 animals prior to the start of Phase II immunizations below (Figure 2).

### Phase II: Lmdd-HIV-gag oral immunization of monkeys with different Lmdd exposure histories

**Figure 3:**
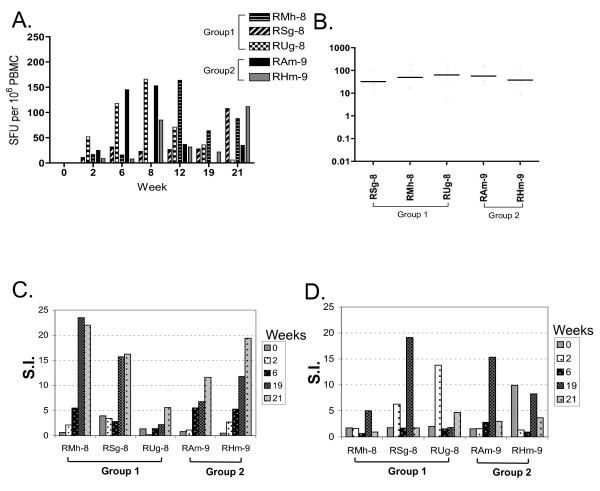
**Gag-specific IFN-gamma-secreting T cells from immunized macaques**. (**A**) PBMC from individual monkeys were tested at the indicated time points for Gag-specific IFN-gamma secreting T cells by *in-vitro *stimulation with overlapping HIV-Gag peptide pools. Vaccinations were given at q.d. x3 at weeks 0, 6, and 19. (**B**) Mean IFN- γ SFU over successive prime and boosting with Lmdd-HIV-gag. No significant differences in ELISPOT generation were observed between groups of naïve rhesus macaques and those having prior oral Lm-vector exposure, P = 0.4 (Wilcoxon rank sum test). (**C**) HIV-Gag specific proliferative responses in Lmdd-HIV-gag-immunized macaques. (**D**) *Listeria *LLO-specific proliferative responses at the indicated time points during vaccination protocol. Stimulation indices (SI) were calculated as described. No significant differences were observed for Gag- or LLO-specific stimulation, P = 0.8 and 0.4 respectively (Wilcoxon rank sum test).

Thirty weeks after the last Lmdd boost (in Group 1 only), we enrolled 2 additional Lm naïve animals (RAm-9, RHm-9). All monkeys then received a series of prime/boost immunizations (q.d. x3) with Lmdd-HIV-gag (Figure 1B) and ELISpots were measured at multiple time points as described. Briefly, PBMC were washed in supplemented RPMI media and seeded onto plates (5 × 10^6 ^cells/ml) in the presence or absence of HIV-1 HXB2-Gag overlapping peptides (NIH AIDS Research and Reference Reagent Program) or Con A. After overnight incubation, cells were removed and plates were incubated with biotinylated anti-IFN-γ antibody (BD Biosciences), followed by incubation with anti-biotin antibody labeled with enzyme. Spots were counted by Immunospot software (BD Biosciences). Two weeks after receiving oral priming with Lmdd-HIV-gag, all five animals showed weak Gag-specific IFN-γ ELISpot responses. Background spots from medium-only wells were subtracted from the wells with peptide stimulation. Wells were considered positive when 3× more spots were found than the average background with a minimum of at least 25 spots and expressed as spot forming units (SFU)/10^6 ^cells. Post-boost, positive ELISpot responses were detectable in most animals. During the course of the three vaccinations, all animals mounted positive IFN-γ ELISpot responses to Gag peptide stimulation, although kinetics of peak responses appeared to differ in each monkey (Figure 3A, B).

Significant Gag-specific proliferative responses (S.I. values >10) were observed in 2 of 3 animals in Group 1, and both Group 2 monkeys (Figure 3C). We also observed significant proliferative responses to LLO peptide stimulation within these animals (Figure 3D). These results demonstrate that oral delivery of attenuated Lmdd-HIV-gag is immunogenic and can induce Gag-specific cellular immune responses, even in the presence of multiple prior Lmdd exposures.

### Anti-vector and anti-HIV Gag antibody responses

To test for the presence of anti-Lm antibodies, an ELISA was employed using whole bacteria (Lm strain 12443) or recombinant LLO as described [17]. Antibody titers are expressed as the end-point dilution that gave an OD value determined as 2 SD above the mean compared to the sera of 6 naïve monkeys. No increases were observed during the course of the immunization in any monkeys (Table 1 and 2). We also screened for anti-Gag IgG responses by using ELISA plates (Fisher Scientific Co, Pittsburgh, PA) coated with 0.5 μg of HIV Gag per well (Immunodiagnostic Inc. Woburn, MA). Only one animal RSg-8, showed a weakly positive Gag-specific titer (data not shown). The lack of significant humoral responses in this model is not surprising; consistent with both our earlier findings [17] and the inability of Lm to elicit potent antibody responses via oral infection routes.

**Table 1:** Serum Anti-Listeria IgG ELISA Titers (whole Listeria).

Groups	Weeks after Lmdd-HIV-*gag *immunization							
Naive	0	6	12	19	21	23	33	34
RAm-9	200	200	200	200	200	200	200	400
RHm-9	200	200	200	200	200	200	200	200
Vector Control								
RSg-8	200	400	400	400	400	400	400	800
RUg-8	200	200	200	400	800	800	800	800
RMh-8	400	400	400	400	800	400	400	400

Lmdd-HIV-gag plasma IgG titers at time points post immunization (0, 6, and 19 weeks). ELISAs were conducted using whole fixed Lm strain 12443, as described [17].

**Table 2:** Serum Anti-Listerial IgG ELISA Titers (rLLO).

Groups	Weeks after Lmdd-HIV-*gag *immunization							
Naive	0	6	12	19	21	23	33	34
RAm-9	200	400	200	200	200	200	200	400
RHm-9	200	400	400	200	200	200	200	200
Vector Control								
RSg-8	200	400	400	400	400	400	400	400
RUg-8	200	200	200	400	800	400	400	400
RMh-8	400	400	400	400	800	400	200	200
								

Anti-Listerial IgG ELISA conducted using recombinant His-tagged Listeriolysin (LLO) as described [17].

### Antigen recall after prolonged rest to orally delivered Lmdd-HIV-gag

Next we sought to determine if any differences exist (between Groups 1 and 2) in anamnestic responses upon re-exposure to Lmdd-HIV-gag. Therefore at thirteen weeks after the last boost, all monkeys were orally dosed using the Lmdd-HIV-gag dose as received previously (Figure 1B). Seven days later, all monkeys were assessed for immune responses to Lm and HIV-Gag.

**Figure 4:**
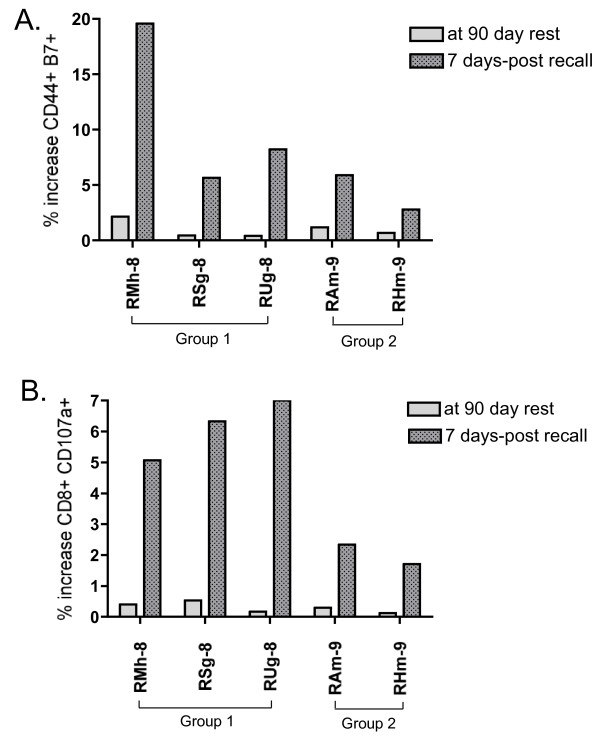
**Expression of homing and degranulation markers in monkeys boosted after prolonged rest**. PBMC were isolated from each animal at the indicated time points following Lmdd-HIV-gag administration and tested for reactivity HIV-Gag peptides. (**A**) Percentage increase in CD44-β7 populations in response to overlapping Gag-peptide. (**B**) Percentage increase in CD8-CD107a populations in response to overlapping Gag-peptide.

We assessed homing of T cells to mucosal sites by following the cell marker CD44 in conjunction with β-7 gut homing marker (BD Biosciences). Upon Gag peptide stimulation, double-positive T cells were increased in all five vaccinees. All five monkeys had at least 5% of the total PBMC population that expressed both markers upon Gag peptide stimulation. Monkey RMh-8 had an unusually high response of nearly 20% of T cells expressing both markers (Figure 4A).

We also determined the relative cytotoxic T lymphocyte (CTL) activity by CD8^+^CD107a^+ ^staining (BD Biosciences). We observed a significant difference between groups 1 and 2 despite a relatively small sample size (Figure 4B). The former group displayed a larger average increase in CTL potential that may be associated with the increased number of Lm exposures. Alternatively, the demonstrated increase in double positive cell percentages could be due to significant levels of bystander T cell activation, or other cells populations, that has been described in murine models of Lm infection [18]. Alternatively, differences in genetic backgrounds between the two groups may account for the observation.

### Continued safety assessment

No adverse clinical effects were observed in any vaccinees during the course of the immunizations. Hematological values and liver chemistries were unremarkable at all time points. These results demonstrated that oral inoculation of live attenuated Lmdd and i.v. D-ala administration was safe and well tolerated in rhesus macaques. Liver toxicity secondary to bacterial invasion can be a serious complication of Lm infection. To assess Lmdd-HIV-gag infiltration into the liver, tissue sections were tested for recombinant Lm harboring the HIV-gag expression cassette. Liver sections were collected (7 days after vaccination), and homogenized in RPMI without antibiotics. Homogenates were clarified then plated in triplicate onto BHI agar plates supplemented with D-ala, erythromycin and streptomycin. Plates were incubated at 37°C for 72 h prior to enumeration of Lmdd-gag colonies. Lmdd-HIV-gag was not found in the liver at 7-days post-inoculation, as measured by plating on selective media specific for recombinant Lmdd-HIV-gag.

For practical reasons, the administration of any candidate HIV vaccine to large populations would be significantly easier if delivered orally. In the present study, we demonstrate in a rhesus model that a live-attenuated Lm vector expressing HIV-*gag *is capable of eliciting Gag-specific responses, even after multiple prior exposures to the vector. Although similar results have been shown in other animal models [12-14], our studies have relied solely on oral delivery. As such, any occurrence of anti-vector immunity might have been increased by multiple dosing using the same route [17]. Despite this potential issue, we observed no difference in Gag-specific ELISpot responses in monkeys with prior Lmdd exposures. Similarly, Lm-vaccine boosting generated modest levels of mucosal homing markers on peripheral blood CD8^+ ^T cells.

While the levels of immunity generated in these animals was certainly not as high as with other vaccines, we believe that at the time of measurement a significant proportion of the response may have been already directed to mucosal sites. Later generation Lm vectors [19-21] may be more effective than providing supplemental D-ala to vaccine preparations. Certainly the ability of Lm to direct immune responses to mucosal regions is an attractive feature of this vector [22]. Thus, this technology should be considered a part of a heterologous prime-boost. Furthermore, the lack of detectable anti-Gag antibodies and low anti-Lm titers, while not unexpected, could be increased by the selection of boost modalities.

The potential benefits of live-vector vaccines must be carefully weighted against safety and toxicity. Wild-type Lm can pose a serious risk for pregnant women, neonates and immunocompromised individuals [3,16,23]. As Lm is ubiquitous, the incidence of exposure to Lm can be from moderate to high within many populations [24], and therefore may pose an obstacle to Lm vaccine development. However, the attenuated vector Lmdd, used in the present study, was shown to be safe in adult and neonatal mice [25]. Similarly, our data show that orally administered Lmdd-HIV-gag was also safe in adult monkeys, indicating limited bacterial invasion into the liver, or complete clearance, by 7 days after boost vaccination.

Our pilot results warrant the testing of attenuated Lm vectors as part of an orally deliverable heterologous prime-boost strategy. However, any future studies should be suitably powered to assess if the current findings are translated to larger populations. We believe that the development of novel next generation Lmdd-based vectors will facilitate that end by increased immunogenicity while retaining a high margin of safety.

## Competing interests

The authors declare that they have no competing interests.

## Authors' contributions

JBW conceived and designed the experiments. FRF produced, titered and quality controlled all Lm vaccine lots. JBW, CCI and LG, participated in performing the ELISPOT assays. JBW and LG performed the proliferative assays. JBW and SM performed the flow cytometric assays. JBW and SYL analyzed the immunology data. RBR performed all ELISA studies. DCA performed the primate work, including tissue sampling and necropsies. JBW and SYL performed statistical analysis. JBW drafted the manuscript. RR and JL revised the manuscript. All authors read and approved the final manuscript.
